# Knowledge, Attitude, and Practices Regarding Vitamin D in Middle-Aged Pakistani Population and the Impact of Sun Exposure on Their Serum Vitamin D Levels

**DOI:** 10.7759/cureus.46034

**Published:** 2023-09-26

**Authors:** Mirza Zeeshan Sikandar, Syed Muhammad Bilal Haider, Iqra Maqbool, Qurat-ul- Ain, Sana Naeem, Awais Naeem, Faheem Usman Sulehri

**Affiliations:** 1 Nephrology, Central Park Medical College, Lahore, PAK; 2 Medicine, Central Park Medical College, Lahore, PAK; 3 Internal Medicine, Akhtar Saeed Medical and Dental College, Lahore, PAK; 4 Community and Family Medicine, Heavy Industries Taxila Education City Institute of Medical Sciences (HITEC-IMS) and Heavy Industries Taxila (HIT) Hospital, Wah, PAK; 5 Internal Medicine, Rashid Latif Medical College, Lahore, PAK; 6 Emergency Medicine, Central Park Teaching Hospital, Lahore, PAK

**Keywords:** pakistan, middle-aged, serum vitamin d levels, sun exposure, practices, attitudes, knowledge, vitamin d

## Abstract

Objective: This study aimed to assess the knowledge, attitude, and practices regarding vitamin D (D-KAP) among middle-aged individuals in Pakistan and examine the impact of sun exposure on their serum vitamin D levels.

Methodology: A cross-sectional study was conducted, involving 80 middle-aged individuals from Lahore, Pakistan. Data on demographics, sun exposure, and D-KAP were collected. Serum vitamin D levels were measured using enzyme-linked immunosorbent assay (ELISA) kits.

Results: The study revealed inadequate knowledge and awareness of vitamin D among the participants. There was a positive correlation between sun exposure duration and serum vitamin D levels, indicating the importance of sun exposure for maintaining optimal vitamin D status. However, there was no significant difference in practice scores between groups, highlighting the need for interventions to bridge the gap between knowledge and practice.

Conclusion: The study emphasizes the need to improve D-KAP in the middle-aged Pakistani population. Public health initiatives should focus on promoting accurate information, addressing cultural beliefs, and encouraging responsible sun exposure practices. Collaborative efforts are crucial to address vitamin D deficiency and its associated health consequences.

## Introduction

Vitamin D, a fat-soluble secosteroid hormone, is a key contributor to musculoskeletal health through its role in calcium and phosphorus homeostasis [1]. Data accrued over the last two decades has shown that low vitamin D levels are associated with a wide range of clinical disorders such as immune dysfunction, cardiovascular diseases, autoimmunity, and neurological deficits, suggesting the involvement of vitamin D in multiple physiological processes [2].

Vitamin D insufficiency affects nearly half of the world’s population. It has been estimated that globally more than one billion people have vitamin D deficiency (VDD). Hypovitaminosis D can, therefore, be dubbed as a condition of pandemic proportions, and it is an independent risk factor for mortality [3]. Owing to ultraviolet-B (UVB)-induced cutaneous synthesis of vitamin D, various lifestyle and environmental factors that reduce exposure are partly responsible for its insufficiency or deficiency. Today the world at large faces a pandemic of hypovitaminosis D with wide-ranging consequences pertaining to general health and wellbeing, spanning all age groups. Over recent years, the seriousness of the condition has gained traction, as established by the rising prescription and self-medication numbers as well as an abundance of research work on the topic [4-7]. Fortified food consumption and its availability also play a fundamental role.

Knowledge, attitude, and practices (KAP) of a region are invaluable to a better understanding of the local prevalence of diseases and disorders. Various research in the east Mediterranean region including Pakistan stipulates a direct correlation between religious and cultural norms and the severity of VDD among the population, especially among women [8-10]. Sun exposure and adequate knowledge of vitamin D play an important role in maintaining normal health and well-being. Data on sun exposure and serum vitamin D in association with their knowledge levels is not available. Therefore, this study is warranted to assess the KAP trends of the general population regarding vitamin D and to assess and correlate serum vitamin D levels with sun exposure and knowledge scores in the local populace.

## Materials and methods

A cross-sectional study design was employed for the present study. Ethical approval (CPMC/IRB-N0/2108) was granted by the institutional review board of Central Park Medical College, Lahore, Pakistan. A total of 80 middle-aged individuals with the age range of 41 years to 47 years were recruited for this study from the general population of Lahore, Pakistan using a random non-convenient sampling method from October 2022 to April 2023. People having skin, liver, kidney, and bone disorders were excluded from this study. Prior written informed consent was obtained from all the participants of the study before enrolment.

Demographic data (age, gender) and anthropometric metrics (height, weight) were recorded on structured study proforma. The body mass index (BMI) of each individual was calculated using the formula weight in kilograms divided by height in meters squared. The enrolled subjects were then grouped into two based on their sun exposure: group 1 having sun exposure less than 1 hour (n=50) and group 2 having sun exposure greater than 1 hour (n=30). For the assessment of daily sun exposure duration, a sun exposure questionnaire scale (SEQS) was employed. The SEQS estimated daily sun exposure by considering the time of the day and exposed body parts and categorizes the sun exposure into three divisions: less than 1 hour/day, 1-2 hour/day, and greater than 2 hours per day [9]. The knowledge, attitude, and practices regarding vitamin D (D-KAP)-38 questionnaire were used for the assessment of D-KAP of the study population. The questionnaire comprised four separate sections, i.e., general knowledge about vitamin D, nutritional knowledge about vitamin D, attitude toward vitamin D, and practice trends about vitamin D. The Likert scale questions of D-KAP-38 in section 1 and section 2 had scores ranging from 0 to 2 and in section 3 and 4, the scoring range was from 1 to 5. It quantifies KAP in terms of scores as general knowledge score ranges from 0 to 22, nutritional scores range from 0 to 10, attitude scores range from 12 to 60, and practice scores range from 10 to 50 [10].

For the assessment of serum vitamin D (25-hydroxy cholecalciferol) levels, 3 mL venous blood was collected from each study participant in lavender-capped blood collection vials (BD Vacutainer; Becton Dickinson) containing potassium-ethylenediaminetetraacetic acid (K-EDTA). The samples were centrifuged within one hour of blood collection at room temperature at a rate of 3000 revolutions per minute for 10 minutes. Immediately after centrifugation, samples were aliquoted and stored at 4°C temperature. Serum vitamin D levels were assessed within 24 hours of blood collection using commercially available enzyme‑linked immunosorbent assay (ELISA) kits (Abbott Architect Chemiluminescent assay) after calibration and standardization.

Statistical analysis

Anonymized data were entered into Microsoft Excel (Microsoft Corporation, Redmond, USA) and checked for errors or duplications. Cross-checked and verified data were then imported into Statistical Package Software for Social Sciences (SPSS) version 26 (IBM Corp., Armonk, NY). Quantitative data was presented as frequencies and percentages. The normality of the quantitative variables was assessed by employing the Shapiro-Wilk test. The median was computed with interquartile range (IQR) for each of the non-normally distributed quantitative variables. Non-parametric Mann-Whitney U test was employed to compare the median between the two groups. Spearman correlation was used to assess the correlation between the study variables. A p-value of less than 0.05 was regarded as significant. 

## Results

The mean age of the study participants was 44.29+14.62 years with no significant age difference in group 1, i.e., sun exposure less than 1 hour/day, and group 2, i.e., sun exposure more than 1 hour/day (43.66+14.20 v/s 45.33+15.49) with a p-value of 0.521. Gender distribution in both groups: group 1 (33 males and 17 females) and group 2 (24 males and 6 females). Incidence of smoking in both groups was assessed; in group 1, 16% (n=8) were smokers, while in group 2, 33.33% (n=10) were smokers. Both groups were also compared and matched for their BMI levels by appliance of the Mann-Whitney U test; 25.80+3.88 kg/m^2^ v/s 26.80+4.39 kg/m^2^ (group 1 v/s group 2) with the p-value of 0.336 (Table 1).

**Table 1:** Comparison of study variables between study groups based on sun exposure

Parameters	Median + IQR		Mann-Whitney U	p-value
	Group 1	Group 2		
Body mass index (kg/m^2^)	25.80+3.88	26.80+4.39	630.5	0.336
25-hydroxy vitamin D (ng/mL)	36.85+19.28	44.10+19.70	530.50	0.048
General knowledge	8.0+9.0	5.0+7.0	530.50	0.012
Nutritional knowledge	4.0+3.0	3.50+4.0	478.00	0.091
Attitude scores	31.50+10.0	31.00+10.0	616.500	0.269
Practice scores	21+12.0	20.00+12.0	569.00	0.112

The normality of the data assessed using Shapiro Wilk test showed the non-parametric distribution of the variables and non-parametric analysis was employed. Significantly, higher serum vitamin D levels were noted in group 2 (having sun exposure for more than 2 hours) with a p-value of 0.048 as narrated in Table 1. Both groups were compared for KAP, and there was no significant difference among study parameters as explained in Table 1.

An overall assessment of D-KAP 38 parameters was made and mean scores for general knowledge (8.55+6.61), nutritional knowledge (3.78+2.45), attitude score (31.40+5.86), and practice scores (20.73+7.41) for the whole study population were noted reflecting lower knowledge attitude and practice scores of whole population.

**Figure 1:**
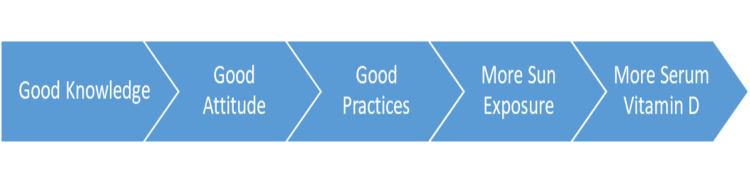
Explaining the correlation between study parameters with one another

On appliance of Spearman correlation, a positive correlation between serum vitamin D and sun exposure was noted with an r-value of 0.241 and p-value of 0.031 suggesting a positive correlation. A positive correlation was also noted between knowledge and attitude scores with an r-value of 0.356 and p-value of 0.001 suggesting higher knowledge leads to a good attitude toward vitamin D. Similarly, a positive correlation was also noted between attitude scores and practice scores with a p-value of 0.665 and p-value of 0.001 suggesting that good attitude leads to good practices toward vitamin D as explained in the flow chart (Figure 1).

## Discussion

VDD is a global health concern with significant implications for various physiological processes and the development of clinical disorders. In this study, we aimed to assess the D-KAP among middle-aged individuals in the Pakistani population and explore the impact of sun exposure on their serum vitamin D levels.

Our findings indicate that the prevalence of VDD is a matter of concern in the middle-aged Pakistani population. The study participants demonstrated relatively low levels of general knowledge, nutritional knowledge, attitude, and practice scores regarding vitamin D. These results are consistent with previous research highlighting the inadequate knowledge and awareness of vitamin D in various populations [11-13]. Insufficient knowledge about vitamin D may contribute to suboptimal sun exposure practices and limited intake of dietary sources rich in vitamin D, ultimately leading to deficiency.

Importantly, our study revealed a positive correlation between sun exposure duration and serum vitamin D levels. Individuals with greater sun exposure (more than 1 hour per day) exhibited higher levels of serum vitamin D compared to those with limited sun exposure (less than 1 hour per day). This finding underscores the vital role of sun exposure in maintaining adequate vitamin D levels, as the cutaneous synthesis of vitamin D through UVB radiation is a primary source for most individuals [14]. However, it is crucial to balance sun exposure to avoid the harmful effects of excessive UV radiation, such as sunburn and an increased risk of skin cancer [15]. Public health initiatives should focus on promoting safe and responsible sun exposure practices while educating individuals about the importance of maintaining optimal vitamin D levels.

Furthermore, our study identified a positive correlation between knowledge and attitude scores, suggesting that a better understanding of vitamin D is associated with more positive attitudes toward its importance. This finding aligns with a previous meta-analysis conducted by Carpenter et al. that has shown a positive association between knowledge and attitudes regarding health-related behaviors [16]. Improving knowledge through targeted educational interventions and awareness campaigns can potentially lead to positive behavior changes and better adherence to recommendations for vitamin D intake and sun exposure.

Interestingly, no significant difference was observed in practice scores between the two study groups based on sun exposure. This may indicate that despite having some knowledge and positive attitudes, individuals may not always translate this knowledge into appropriate practices. Factors such as accessibility to fortified foods, cultural beliefs, and individual preferences might influence the translation of knowledge into actionable practices [17]. Future research and public health strategies should aim to bridge this gap between knowledge and practice to ensure better vitamin D status in the population.

While our study provides valuable insights into the D-KAP levels in the middle-aged Pakistani population, it has certain limitations. The study sample was relatively small and limited to a specific geographic area, which may limit the generalizability of the findings. Additionally, self-reported measures and the use of questionnaires may introduce response bias. Further studies with larger sample sizes and diverse populations are warranted to confirm and expand upon these findings.

## Conclusions

There is a positive correlation between sun exposure duration and serum vitamin D levels, indicating the importance of sun exposure for maintaining optimal vitamin D status. This study highlights the significance of improving D-KAP in the middle-aged Pakistani population. Public health initiatives should focus on providing accurate information, addressing cultural beliefs, and promoting safe sun exposure practices. Collaborative efforts involving healthcare professionals, policymakers, and community stakeholders are crucial in addressing the widespread issue of VDD and its associated health consequences.

## References

[1] Shah SIA, Iqbal S, Sikandar MZ, Qazi Qazi, UY UY, Haq I (2021). Serum vitamin D and cardiometabolic markers: a comparative study in adult men based on body mass index. IIUM Med J Malays.

[2] Iqbal S, Shah SIA, Sikandar MZ (2019). Relationship between serum vitamin D and insulin resistance in normal and overweight or obese men. Professional Med J.

[3] Shah SIA, Sikandar MZ, Qazi UY, Haq I (2021). Comparative assessment of vitamin D and parathyroid hormone as risk factors of myocardial infarction and their correlation with lipid profile. Medical Science.

[4] Alswailmi FK, Sikandar MZ, Shah SIA, Parrey MuR, Jelani S (2021). Serum Vitamin D, sun exposure and clinical attributes of local patients with respiratory allergies. Medical Science.

[5] Arshad S, Zaidi SJ (2022). Vitamin D levels among children, adolescents, adults, and elders in Pakistani population: a cross-sectional study. BMC Public Health.

[6] Mustafa A, Shekhar C (2021). Concentration levels of serum 25-Hydroxyvitamin-D and vitamin D deficiency among children and adolescents of India: a descriptive cross-sectional study. BMC Pediatr.

[7] Mithal A, Wahl DA, Bonjour JP (2009). Global vitamin D status and determinants of hypovitaminosis D. Osteoporos Int.

[8] Rovner AJ, O'Brien KO (2008). Hypovitaminosis D among healthy children in the United States: a review of the current evidence. Arch Pediatr Adolesc Med.

[9] Patwardhan VG, Mughal ZM, Chiplonkar SA (2018). Duration of casual sunlight exposure necessary for adequate vitamin D status in Indian men. Indian J Endocrinol Metab.

[10] Amiri P, Asghari G, Sadrosadat H, Karimi M, Amouzegar A, Mirmiran P, Azizi F (2017). Psychometric properties of a developed questionnaire to assess knowledge, attitude and practice regarding vitamin D (D-KAP-38). Nutrients.

[11] Al-Shaikh GK, Alzaheb RA, Al-Amer OM, Alshowair MA, Farooq MU (2019). Knowledge and attitudes toward vitamin D and its association with vitamin D levels among Saudi Arabian residents. J Nutr Metab.

[12] AlBishi LA, Prabahar K, Albalawi YM (2018). Knowledge, attitude and practice of health care practitioners in Saudi Arabia, with regard to prevention of vitamin D deficiency in infancy. Saudi Med J.

[13] Almutairi KM, Al-Mutairi A, Al-Saedi AJ, Alsabaani A (2019). Assessment of knowledge, attitudes, and practices of vitamin D among the Saudi population. J Pharm Bioallied Sci.

[14] Holick MF (2004). Sunlight and vitamin D for bone health and prevention of autoimmune diseases, cancers, and cardiovascular disease. Am J Clin Nutr.

[15] Green AC, Williams GM, Logan V, Strutton GM (2011). Reduced melanoma after regular sunscreen use: randomized trial follow-up. J Clin Oncol.

[16] Carpenter CJ (2010). A meta-analysis of the effectiveness of health belief model variables in predicting behavior. Health Commun.

[17] Quaidoo EA, Williams JE, Steffen LM (2014). Knowledge, attitude, and practices related to calcium and vitamin D intake among adult patients with chronic kidney disease. J Ren Nutr.

